# Comparison of Biological Properties of Two Medicinal Extracts of the Tehuacan-Cuicatlan Valley

**DOI:** 10.1155/2018/4918090

**Published:** 2018-11-13

**Authors:** Karla S. Martinez-Elizalde, Marco A. Rodriguez-Monroy, Cesar M. Flores, Luis B. Hernandez-Portilla, Elizabeth Barbosa-Cabrera, Ma. Margarita Canales-Martinez

**Affiliations:** ^1^Doctorado en Investigacion en Medicina, Escuela Superior de Medicina (ESM), Instituto Politecnico Nacional (IPN), CDMX, Mexico; ^2^Carrera de Medicina, Facultad de Estudios Superiores-Iztacala UNAM, Tlalnepantla, Edo. Mex., Mexico; ^3^Laboratorio de Fisiologia Vegetal, UBIPRO Laboratorio Nacional en Salud. Facultad de Estudios Superiores-Iztacala UNAM, Tlalnepantla, Edo. Mex., Mexico; ^4^Escuela Superior de Medicina, IPN, Casco de Santo Tomas, Miguel Hidalgo, Mexico, D.F., Mexico; ^5^Laboratorio de Farmacognosia, UBIPRO Facultad de Estudios Superiores-Iztacala UNAM, Tlalnepantla, Edo. Mex., Mexico

## Abstract

In the Valley of Tehuacan-Cuicatlan,* Cyrtocarpa procera* and* Bursera morelensis* are located and are used in traditional medicine. In this research, several biological properties were evaluated. The methanol extracts of* C. procera* (MeCp) and* B. morelensis* (MeBm) were obtained by maceration. The antibacterial activities of the extracts were evaluated by the Kirby–Baüer disc-diffusion method. The wound healing activity was evaluated by histopathological analysis. Both extracts had a bacteriostatic effect in the* Staphylococcus aureus* (MeCp MIC = 0.25 mg/mL and MeBm MIC = 1 mg/mL) and the* Vibrio cholerae* (MeCp MIC = 1 mg/mL and MeBm MIC = 4 mg/mL). Both extracts demonstrated a wound healing efficacy similar to the reference standard (Recoveron). They also showed a high antioxidant capacity (MeCp SC_50_ = 5.75 *μ*g/mL and MeBm SC_50_ = 4.27 *μ*g/mL). These results are related to the concentration of phenols (MeCp = 166 and MeBm = 236.6 mg GAe/g) and flavonoids of MeCp = 16 and MeBm = 22 *μ*g Qe/g. Both extracts, acting in a similar way in microorganisms that cause infection thanks to their antioxidant activity, favor the healing of wounds. This is the first study in which the biological properties of these two species are compared.

## 1. Introduction

The Valley of Tehuacan-Cuicatlan is one of the regions of great interest, located in one of the more excellent biotic reserves of Mexico. In this area, there are approximately 2700 species of plants. According to these numbers, the valley is probably an area with a great biodiversity within the barren zones of North America [1].

Between 10 and 11.4% of the Mexican flora thrive in this zone, with 365 endemic species that represent 13.9% of the total species in this area, while different human communities that inhabit the region use more than 25% of the plants in the valley [2]. Canales et al., in an ethnobotanical study, showed a list of species used by the inhabitants of the region to relieve various ailments and mentioned that about 46 species have a medicinal use, including the bark of* Cyrtocarpa procera* Kunth and the bark of* Bursera morelensis* Ramirez [3].

*Cyrtocarpa procera* Kunth is an indigenous Mexican tree that belongs to the Anacardiaceae plant family. This species is used in traditional Mexican medicine, where it is known locally as “chupandilla” or “copalcojote.” The bark is employed in infusions to treat ailments, such as diarrhea, dysentery, coughs, and wound healing. This species is endemic to Mexico and is distributed in the center of the country (Colima, Michoacan, Estado de Mexico, Oaxaca, Morelos, Jalisco, Nayarit, Guerrero, and Puebla states) [3–5]. The biological properties of* C. procera* bark have been poorly studied, phytochemical studies have reported the isolation and identification of some sterols and the fatty acids in the hexanic extract [6, 7]. In addition, the antibacterial activity of the methanolic extract of the bark and fruit of* C. procera* has been described [3, 8] and the anti-*Helicobacter pylori* activity of aqueous and methanolic extracts as well as the antiulcer activity of* C. procera* [9, 10].

Moreover,* B. morelensis* is an endemic species of Mexico that belongs to the Burseraceae plant family. Its bark is used in traditional Mexican medicine, employed to treat various ailments such as pain stomach aches, cold sores, wound infections, and pimples; it is known as “copalillo,” “red cuajiote,” “red stick,” or “mulatto stick” [11]. The essential oil of Burseraceae family has been shown to have antibacterial activity [12, 13], anti-inflammatory activity [14] and cytotoxic activities [15, 16]. On the other hand, the extracts of some Bursera species have anti-inflammatory activities [17, 18]. With respect to* B. morelensis*, several studies have described the antibacterial activity of the methanolic extract [3] and the anti-inflammatory, analgesic, and antioxidant properties of the methanolic extract bark [19].

However, the biological properties of these species have not been compared to each other and, in this work, the wound healing activity is reported for the first time.

## 2. Materials and Methods

### 2.1. Plant Material

The* C. procera* and* B. morelensis* barks were collected in August 2012 in San Rafael, Coxcatlan, Puebla, and the botanical authentication of the specimen was done by M. C. Maria Edith Lopez Villafranco (curator at the IZTA Herbarium). Voucher specimens were deposited in the IZTA herbarium at the Facultad de Estudios Superiores Iztacala (voucher no. 2412 IZTA and no. 42123 IZTA, respectively). The specimens were collected in the field with permission from the “Secretaria de Medio Ambiente y Recursos Naturales” (SGPA/DGVS/1266).

San Rafael is a village in the municipality of Coxcatlan, which is located to the southeast of the Tehuacan-Cuicatlan Valley at 18°12' and 18°14' North and 97°07' and 97°09' West, residing 957 m above sea level. The climate is dry or arid with summer rains and a mean temperature of 22°C [20].

### 2.2. Preparation of the Extracts

The extract of the* C. procera* bark (1009 g) and* B. morelensis* bark (1200 g) was obtained through maceration exhaustive with methanol (1:10 w/v) at room temperature. After filtration, the solvent was evaporated under reduced pressure, generating the methanolic extract of each species (MeCp and MeBm, respectively). After removing the solvent, the dry weight yield was obtained, 131.59 g (29.51%) of MeCp and 236 g (19.66%) of MeBm.

### 2.3. Antibacterial Activity

Bacteria strains are* Streptococcus mutans* ATCC 35668,* Streptococcus mutans* clinical case,* Staphylococcus aureus* clinical case,* Staphylococcus aureus* ATCC 25923,* Staphylococcus aureus* methicillin resistant,* Staphylococcus epidermidis* clinical case,* Staphylococcus epidermidis* ATCC 12228,* Enterococcus faecalis* clinical case,* Enterococcus faecalis* ATCC 29212,* Actinomyces viscosus* clinical case,* Pseudomonas aeruginosa* clinical case,* Pantoea agglomerans* clinical case,* Enterobacter cloacae* clinical case,* Escherichia coli* clinical case 1,* Escherichia coli* clinical case 2,* Vibrio cholerae* clinical case, and* Vibrio cholerae* (a clinical isolate corresponding to group 01 that produces enterotoxin and has the “Inaba” serotype and “El Tor” biotype).

The antibacterial activity was measured through the Kirby–Baüer disc-diffusion method [21]. We followed the methods of Martinez-Elizalde et al. [8]. The microorganisms were grown overnight at 37°C in 10 mL of Müeller Hinton broth (Bioxon 260-1, Estado de Mexico, Mexico). The cultures were adjusted to turbidities comparable to that of a Mc Farland no. 0.5 standard with a sterile saline solution. Petri dishes containing Müeller Hinton agar (Bioxon, Edo. de Mexico, Mexico) were impregnated with these microbial suspensions. Subsequently, 200 mg/mL solutions of each extract were prepared, and 5 mm discs (Whatman no. 5) were impregnated with 10 *μ*L of each extract (final doses per disc: 2 mg of MeCp and MeBm). Discs impregnated with 10 *μ*L of methanol were used as negative controls. Discs containing chloramphenicol (25 *μ*g) were used as positive controls. The plates were incubated overnight at 37°C, and the diameter of any resulting inhibition zones (mm) was measured. Each experiment was repeated at least three times. The minimal inhibitory concentration (MIC) was estimated using the broth dilution method [21]. Diluted plant extracts (8.0 to 0.125 mg/mL) were used. The tubes were inoculated with a 1 × 10^5^ CFU/mL microorganism suspension. The MIC values were defined as the lowest extract concentration that prevents visible bacterial growth after 24 h of incubation at 37°C. Each experiment was repeated at least three times. The bactericidal kinetic assay was performed using the appropriate concentrations of the extract (corresponding to MIC_50_, MIC, and MBC) [22], each experiment was repeated at least three times.

### 2.4. Wound Healing Activity

#### 2.4.1. Animals

Male CD-1 mice (20–25 g) were purchased from Harlan Laboratories (Mexico). The mice were maintained in a pathogen-free environment at the FES Iztacala Bioterium. All studies were conducted in accordance with the “Guide for the Care and Use of Laboratory Animals” of the National Institute of Health [23] and they were handled following the animal care guidelines in accordance with regulations enacted by the Federal Government of Mexico (NOM-062-ZOO-1999) [24]. An internal committee reviewed the protocol for the care of laboratory animals. Mice were sacrificed using a CO_2_ chamber and all efforts were made to their minimize pain.

#### 2.4.2. Experimental Groups

For the incision wound model, the animals were divided into five groups (n=5 per group) as follows: group I: normal skin; group II: positive control (Recoveron-N®); group III: untreated; group IV: MeCp 10%; and group V: MeBm 10%. For the preparation of the experimental treatments, 1 g of each extract was dissolved separately in 10 mL of the vehicle (1% of Gel carbomer 940 in deionized water).

#### 2.4.3. The Incision Wound Model

The dorsal fur of the animals was shaved with an electric clipper and their hair was removed with Veet® depilation cream; the incision model mice were anaesthetized by isoflurane (5%) [25]. The longitudinal incision was made using a bistoury for 1 cm in the skin in the dorsal region. The injury covered the epidermis, dermis, and hypodermis, as described by Vaisberg et al. [26]. The formulation ointment and standard drug (0.5 mL) were applied twice a day for 10 days, until wound closure.

*(1) Histopathological Analysis.* After 10 days of treatment, the injury regions were zinc-fixed and embedded in paraffin. Then, 5 micrometer thick tissue sections were prepared and stained with hematoxylin and eosin (H&E) to evaluate the wound healing. Histopathological changes in the section of the wound healing were observed under the microscope (Zeiss Scope; A1 with camera axiocam ICc 5) at 10X magnification.

### 2.5. DPPH Scavenging Capacity

We followed the methods of Martinez-Elizalde et al. [8], the ability of the extracts to quench free radicals was evaluated using the spectrophotometric method at 517 nm by decolorizing a methanolic DPPH solution, as described by Murillo [27]. A freshly prepared DPPH solution (4 mg/100 mL methanol) was used during the assays. The samples were dissolved in methanol (1, 2, 4, 6, 8, 10, 15, 20, 25, 30, 35, 40, 45, 50, 60, 70, 80, 90, and 100 *μ*g/mL), and the DPPH solution was the control. The degree of discoloration reveals the free radical scavenging efficiency of the samples. Quercetin was used as a reference free radical scavenger (SC_50_ = 4.6 *μ*g/mL). The percentage of DPPH discoloration was calculated as follows:

  Inhibition Percentage (Ip) = [(AB − AA)/AB)] × 100

where AA is the absorbance of the sample and AB is the absorbance of the control [28].

The SC_50_ was calculated when allowed according to the scavenging efficiency. The SC_50_ values were calculated through rectangular hyperbola regressions, where the abscissa represented the concentration of the tested plant extract and the ordinate represented the average percentage of the scavenging capacity from three replicates.

### 2.6. Antioxidant Activity Index (AAI)

The antioxidant activity index (AAI) is calculated as follows:

  AAI = DPPH (*μ*g/mL)/SC_50_ (*μ*g/mL)

where DPPH (*μ*g/mL) is the final concentration of DPPH^•^ solution and SC_50_ (*μ*g/mL) is the concentration providing DPPH Scavenging Capacity.

The following criteria of AAI values were established for plant extracts: poor activity < 0.05 < moderate < 1.0 < strong < 2.0 < very strong [29].

### 2.7. Determination of Total Phenolics

The total phenols were determined by the Folin Ciocalteu reagent [30]. A solution of 1 mL of methanol extract (0.05 mg/mL) was mixed with distilled water (7 mL), Folin Ciocalteu reagent (0.5 mL), and 1.5 mL of Na_2_CO_3_ (20%), was repeated at least three times. The mixtures were allowed to stand for 120 min in the dark and the total phenols were determined using a spectrophotometer at 760 nm. The standard curve was prepared using 0.00625, 0.0125, 0.025, 0.05, 0.1, and 0.2 mg/L solutions of gallic acid in water. However, the total phenol values are expressed in terms of gallic acid equivalent (GAe/g of extract).

### 2.8. Determination of Total Flavonoids

The total flavonoids were determined by the Dowd method [31]. A mixture solution of 2% aluminum trichloride (AlCl_3_) in HPLC grade methanol with a concentration of 0.2 mg/mL was used. After 10 minutes, readings were taken at 415 nm using a spectrophotometer. The calibration curve was prepared using 1–100 *μ*g/mL of quercetin. The total flavonoids were expressed as mg of the quercetin equivalent (Qe/g of extract). Each determination was repeated at least three times.

### 2.9. High-Performance Liquid Chromatography

The chemical compounds were identified using HPLC (Hewlett Packard 1100 system equipped with a diode array detector-DAD). The analysis was performed with an Allphere ODS-1 C18 column (250 × 46 mm, 5 *μ*m) at 23°C. The flow rate was as follows: 1 mL/min for 0–5 min; 1.5 mL/min for 5–7.5 min. The mobile phase was isocratic (MeOH: Acetonitrile: H_2_O (25:25:50). The analysis of the peaks of the chromatogram was determined with ultraviolet (UV) spectra (280 nm) using the Chemstation A.09.03 software.

### 2.10. Statistical Analysis

All experiments were performed in triplicate. The means and standard deviations of the experiments were determined. The SC_50_ values were calculated through a rectangular hyperbola model. The statistical differences between the treated and the control groups were evaluated through an analysis of two-way variance (ANOVA). Values of p ≤ 0.05 were considered to be statistically significant. The calculations were performed using the GraphPad Prism software (version 6.0; GraphPad Software Inc., San Diego, CA, USA).

## 3. Results

### 3.1. Antibacterial Activity

The results of the antibacterial activity of MeCp and MeBm are shown in Table 1. The MeCp and MeBm are active against Gram-positive and Gram-negative bacteria. The MeBm is not active in the* P. aeruginosa* clinical case and the* P. agglomerans* clinical case. There are no significant differences (*p* ≤ 0.05) when comparing the activity of both extracts; however, when making a comparative analysis on the sensitivity of the bacterial group, there are significant differences (*p* ≤ 0.05) in Gram-negative bacteria. The MeCp has a higher activity compared to MeBm.

Regarding the results on the MIC of both extracts, it was observed that both extracts exhibited the lowest MIC in the* S. aureus* clinic case (MIC = 0.5 mg/mL MeCp and MIC = 1 mg/mL MeBm) and the* V. cholera* clinical case (MIC = 1 mg/mL MeCp and MIC = 4 mg/mL MeBm) (Table 1).

**Figure 1:**
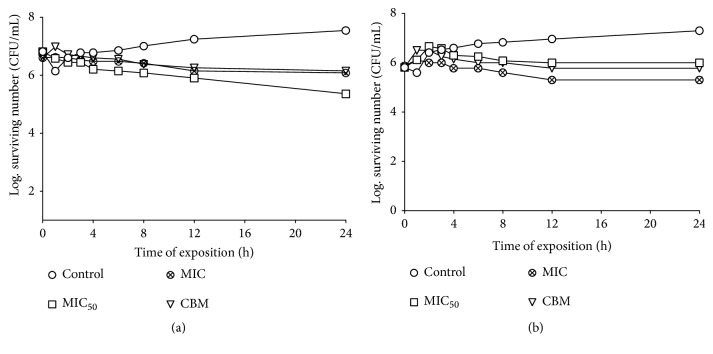
(a) The survival curve for the* Staphylococcus aureus *clinical case exposed to MeCp. The concentrations were 0.25 mg/mL (MIC_50_), 0.50 mg/mL (MIC), and 6.00 mg/mL (MBC); (b) the survival curve for* Vibrio cholerae* exposed to MeCp. The MeCP was added to each experimental culture at the time of 0 s. The concentrations were 0.50 mg/mL (MIC_50_), 1.00 mg/mL (MIC), and 6.00 mg/mL (MBC); the MeCp was added to each experimental culture at the time of 0 s. The control tube did not contain MeCp.

Figures 1(a) and 1(b) show the effects of MeCp on the survival curves against the* S. aureus* clinic case and the* V. cholera* clinical case. The minimum inhibitory concentration (MIC_50_ = 0.25 mg/mL) had a bacteriostatic effect and similar activity was shown against the* V. cholera* clinical case (MIC = 1 mg/mL).

### 3.2. Wound Healing Activity

The wound healing activity was shown in the histopathological analysis (Figure 3). In the group of normal skin, three defined structures (epidermis, dermis, and hypodermis), epidermal appendages (hair follicles, sweat glands), and muscle tissue were observed (Figure 3(a)).  Figure 3(b) shows the results of the positive control where it was observed that the scab remains were still present, the epidermis was thickened due to the mitotic activity of the basal cells, and the dermis was observed as being dense due to the activity of the fibroblasts. Additionally, blood vessels were observed but no epidermal adnexa were observed, so the images suggest that they are probably of the proliferative stage.  Figure 3(c) corresponds to the untreated group and it was observed that, although the wound has already contracted, there was still an inflammatory infiltrate and the thickness of the epidermis was scarce.  Figure 3(d) corresponds to the treatment with 10% of MeCp; a great production of blood vessels was observed, as well as the thickened epidermis and the dense dermis in remodeling. Finally, Figure 3(e) shows the treatment group of 10% MeBm where the keratin layer is thickened. Additionally, there are blood vessels and the dermis are dense in the remodeling.

### 3.3. Antioxidant Capacity

With respect to at the antioxidant activity, there were no significant differences (*p* ≤ 0.05) between the extracts. As the MeCp has an SC_50_ of 5.75 *μ*g/mL and the MeBm has an SC_50_ of 4.27 *μ*g/mL, the positive control quercetin has an SC_50_ of 4.6 *μ*g/mL.

### 3.4. Antioxidant Activity Index (AAI)

With respect to the AAI, the MeCp has an AAI of 5.22 and MeBm has an AAI of 7.03. Thus, both extracts present indexes of very strong antioxidant activity according to the Scherer and Godoy criterion [29].

### 3.5. Determination of the Total Phenolics and Total Flavonoids

The previous results are related to the concentration of phenols (MeCp = 166 mg GAe/g and MeBm = 236.6 mg GAe/g) and flavonoids (MeCp = 16 *μ*g Qe/g and MeBm = 22 *μ*g Qe/g) contained in the extract.

### 3.6. High-Performance Liquid Chromatography

Using different flavonoid standards, only catechin could be identified for both extracts by HPLC analysis, for the MeCp = 2.449 min (Figure 4(a)) and for the MeBm = 2.452 min (Figure 4(b)).

## 4. Discussion

The inhabitants of the Tehuacan-Cuicatlan Velley mentioned that about 46 species have a medicinal use, including the bark of* Cyrtocarpa procera* and the bark of* Bursera morelensis*, which are used separately in infusions to treat conditions such as diarrhea, dysentery, cough, and wound healing among other ailments [3–5]. In this work, the biological properties of these two species were studied and compared.

The biological properties that are studies in this work are the antibacterial activity, antioxidant capacity, and wound healing activity; various scientific studies have reported that herbal plants are a good source of antioxidant and antimicrobial compounds and play a wide role in the treatment of the chronic wound infections [32, 33].

MECp and MEBm presented activity against Gram-positive and Gram-negative bacteria (Table 1). The results agree on the ethnobotanical use of both species for the treatment of various diseases [3, 4, 6]. Although there were no significant differences (*p* ≤ 0.05) when comparing the activity of both extracts, MeCp have a greater activity compared to MeBm in the group of Gram-negative bacteria. This result is very interesting because the Gram-negative bacteria have a membrane that is more resistant to organic solvents and less permeable to molecules with a hydrophobic nature that makes them more resistant [34]. These results coincide with the antibacterial activity using the extract of the fruit from* C. procera *[8] and with the activity on* Helicobacter pylori* using the bark extract of* C. procera* [10]. With respect to the antibacterial activity of MeBm, it has been known that the essential oil has an activity against pathogenic bacteria, in particular, Gram-positive bacteria [35], as found in the present study.

**Figure 2:**
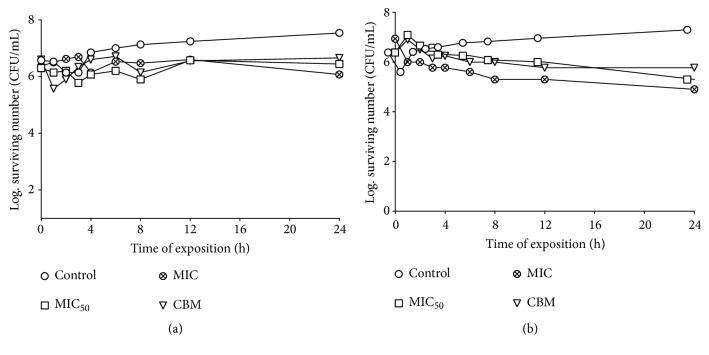
(a) The survival curve for the* Staphylococcus aureus *clinical case exposed to MeBm. The concentrations were 1.00 mg/mL (MIC_50_), 4.00 mg/mL (MIC), and 8.00 mg/mL (MBC); (b) the survival curve for the* Vibrio cholerae* clinical case exposed to MeBm. The concentrations were 2.00 mg/mL (MIC_50_), 4.00 mg/mL (MIC), and 8.00 mg/mL (MBC). The MeBm was added to each experimental culture at the time of 0 s. The control tube did not contain MeBm.

Bacteria such as* S. aureus* and some* Vibrio* species are associated in the areas of injury [36, 37], so we evaluated the effect on the growth curve of the extracts against these types of bacteria. The MeCp and MeBm have a bacteriostatic effect on the* S. aureus* clinical case and the* V. cholera *clinical case. Both extracts decrease the bacterial population when using low concentrations (MeCp MIC_50_ = 0.25 mg/mL and MIC = 1 mg/mL, Figures 1(a) and 1(b); MeBm MIC = 1 mg/mL and MIC = 4 mg/mL MeBm, Figures 2(a) and 2(b)). This observation is very important because these results indicate that the activity of these extracts is not dose-dependent as the best concentration for the bacteriostatic effect is MIC_50_ and, thus, the immune system of the host can respond against these pathogens.

**Figure 3:**
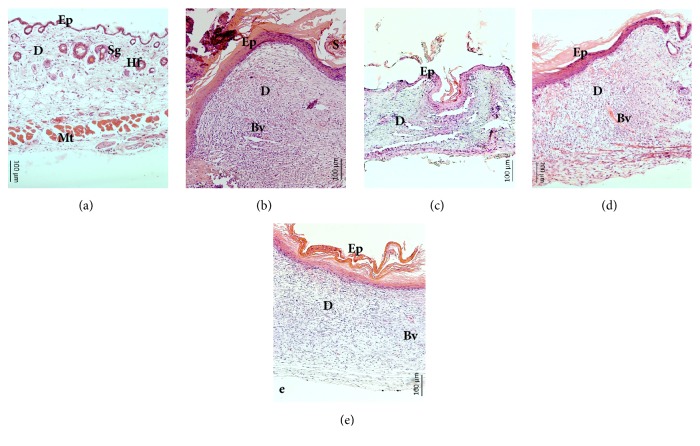
Wound healing histology for each group, 10 days after surgery. (a) group I: normal skin; (b): group II: positive control (Recoveron-N®); (c): group III: untreated; (d): group IV: MeCp 10%; and (e): group V: MeBm 10%. Hematoxylin & Eosin staining shows the collagen fibers stained pale pink, the cytoplasm stained purple, the nuclei stained blue, and the red blood cells stained cherry red at 10X magnification. Bar = 100 *μ*m, Ep = Epidermis, D = dermis, Mt = Muscular tissue, Sg = Sebaceous gland, Hf = Hair follicle, Bv = Blood vessel, and S = Scab.

**Figure 4:**
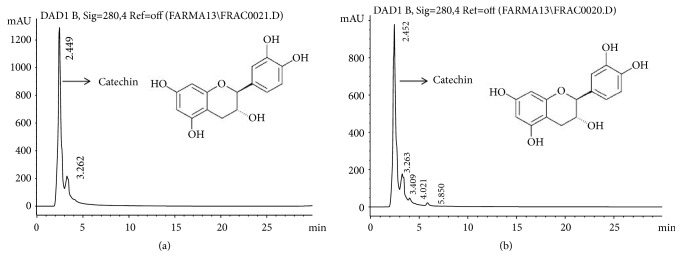
Chromatograms of HPLC analysis. (a) Chromatogram of MeCp, (b) Chromatogram of MeBm. HPLC analysis with C18 column at 23°C and UV detector at 280 nm using the Chemstation A.09.03 software. The mobile phase was isocratic (MeOH: Acetonitrile: H_2_O (25:25:50). Chromatograms show catechin.

According to the wound healing activity, MeCp and MeBm show anti-inflammatory activity since, in the histological sections, a scarce inflammatory infiltrate was observed (Figures 3(d) and 3(e)). In addition, after 10 days of treatment, both extracts favored the production of blood vessels and the synthesis of collagen and elastin by fibroblasts; so, it could be said that they are in the proliferative phase. Comparing the effect of Recoveron-N® with the other treatments, it was observed that they were in the same phase, with the exception that the treatments no longer have a scab (Figures 3(d) and 3(e)). Similar effects were reported for* C. procera* in healing gastric ulcers [7, 10]; however, this is the first work reporting the wound healing activity of this medicinal species.

On the other hand, for* B. morelensis*, there were reports on the biological properties involved in the healing process whereby its anti-inflammatory and antioxidant activity could favor the healing process [19]. Additionally, the essential oil and exudates of* Bursera spp* exhibit anti-inflammatory activity [14, 17, 35]. However, this is also the first report on wound healing activity using the incision model.

The MeCp have an SC_50_ of 5.75 *μ*g/mL and the MeBm have an SC_50_ of 4.27 *μ*g/mL. Comparing these results, there are no significant differences (*p* ≤ 0.5) between the extracts. The antioxidant activity of the positive control quercetin has an SC_50_ of 4.6 *μ*g/mL. Thus, both extracts have very good antioxidant capacities, not only because the value of SC_50_ is very similar to quercetin, but also because both extracts have a very strong antioxidant activity index.

The quantifications of the total phenols and total flavonoids are related to the antioxidant capacity. From the concentrations of the phenols (MeCp =166 mg GAe/g and MeBm=236.6 mg GAe/g) and the flavonoids (MeCp=16 *μ*g Qe/g and MeBm= 22 *μ*g Qe/g) contained in the extract, using an HPLC analysis, only catechins could be identified for both extracts (MeCp = 2.449 min and MeBm = 4.452 min). The antioxidants, upon interaction with DPPH, transfer either an electron or a hydrogen atom to DPPH, thus, neutralizing its free radical character [38]. The flavonoids have been demonstrated to have anti-inflammatory, antiallergenic, antiviral, antiaging, and anticarcinogenic activities. The broad therapeutic effects of flavonoids can be largely attributed to their antioxidant properties. In addition to an antioxidant effect, the flavonoid compounds may exert protection against heart disease through the inhibition of cyclooxygenase and lipoxygenase activities in platelets and macrophages [39]. For the flavonoids and their derivatives, those with the strongest antioxidant potential include delphinine, epicatechin, kaempferol, quercetin, and luteolin. Quercetin, the most abundant dietary flavonol, is a potent antioxidant because it has all structural features for free radical scavenging activity [40, 41]. It has been reported that catechins interact with the cell wall and membrane of bacterial cells [42–44]. It has been reported that the hydroxyl groups of Epigallocatechin gallate (EGCg) interact with the nitrogen atom of the side chains of basic amino acids through the hydrogen bonds of the cell membrane and with the arginine residues (Arg) located in the inner wall of the cell with the porin cylinder through the hydrogen bonds destabilizing the membrane, making it more permeable [45].

According to the data obtained, in the present study both extracts have similar biological properties such as antibacterial activity, antioxidant capacity and wound healing activity, probably being phenols and catechin the compounds that are intervening in these biological properties.

## 5. Conclusions

This research agrees with the ethnobotanical use of* Cyrtocarpa procera* (MeCp) and* Bursera morelensis *(MeBm), acting in a similar way against microorganisms that cause infections thanks to the antioxidant activity they have, thus favoring the healing of wounds.

## Figures and Tables

**Table 1:** The antibacterial activity of the *Cyrtocarpa procera* and *B. morelensis* bark.

Bacteria	Positive Control Chloramphenicol (Inhibition Halos mm)	MeCp		MeBm	
		(Inhibition Halos mm)	CMI (mg/mL)	(Inhibition Halos mm)	CMI (mg/mL)
Sm 35668	33.00 ± 1.00	7.33 ± 0.57	0.25	9.33 ± 0.57	0.25
Sm	30.00 ± 1.00	8.00 ± 0.00	0.50	8.66± 0.57	0.50
Sa	28.00 ± 0.00	8.33 ± 0.57	0.50	8.66± 0.57	1.00
Sa 25923	19.66 ± 0.57	7.00 ± 0.00	0.50	8.00 ± 0.00	4.00
Sa mr	19.66 ± 1.52	9.00 ± 0.00	1.00	9.33 ± 0.57	2.00
Se 35984	29.00 ± 0.00	8.66 ± 0.57	1.00	10.33 ± 0.57	2.00
Se	30.30 ± 0.57	10.67 ± 0.57	0.50	11.66 ± 0.57	2.00
Se 12228	25.33 ± 1.52	9.66 ± 0.57	0.50	10.00 ± 0.00	2.00
Ef	27.33 ± 1.52	9.66 ± 0.57	1.00	8.00 ± 0.00	1.00
Ef 29212	24.30 ± 0.57	10.00 ± 0.00	0.125	9.66 ± 0.57	0.50
Av	24.66 ± 1.15	7.76 ± 0.57	0.50	8.66 ± 0.57	2.00
Pa	21.33 ± 0.57	8.66 ± 0.57	8.00	Na	Na
Pag	20.0 ± 0.00	9.33 ± 0.57	1.00	Na	Na
Ecl	23.00 ± 0.00	9.33 ± 0.57	2.00	10.00 ± 0.00	1.00
Ec1	20.00 ± 0.00	9.00 ± 0.00	8.00	9.66 ± 0.57	4.00
Ec12	21.67 ± 1.70	11.66 ± 0.57	1.00	13.00 ± 0.00	4.00
Vch	23.3 ± 1.00	8.00 ± 0.00	1.00	9.66 ± 0.57	4.00
Vch Tor	25 ± 0.00	8.66 ± 0.57	4.00	9.00 ± 0.00	2.00

Sm: *Streptococcus mutans* ATCC 35668; Sm: *Streptococcus mutans* clinical case; Sa: *Staphylococcus aureus* clinical case; Sa 25923: *Staphylococcus aureus* ATCC 25923; Sa mr: *Staphylococcus aureus* methicillin resistant; Se 35984: *Staphylococcus epidermidis* ATCC 35984; Se: *Staphylococcus epidermidis* clinical case; Se 12228: *Staphylococcus epidermidis* ATCC 12228; Ef: *Enterococcus faecalis* clinical case; Ef 29212: *Enterococcus faecalis* ATCC 29212; Av: *Actinomyces viscosus* clinical case; Pa: *Pseudomonas aeruginosa* clinical case; Pag: *Pantoea agglomerans* clinical case; Ecl: *Enterobacter cloacae* clinical case; Ec1: *Escherichia coli* clinical case 1; Ec2: *Escherichia coli* clinical case 2; Vch: *Vibrio cholerae* clinical case; Vch Tor: *Vibrio cholerae* (a clinical isolate corresponding to group 01 that produces enterotoxin and has the “Inaba” serotype and “El Tor” biotype). Na: no activity.

## Data Availability

The data used to support the findings of this study are included within the article.
